# The impact of COVID-19 on pregnancy outcomes in a diverse cohort in England

**DOI:** 10.1038/s41598-022-04898-5

**Published:** 2022-01-18

**Authors:** Michael Wilkinson, Edward D. Johnstone, Louise E. Simcox, Jenny E. Myers

**Affiliations:** 1 Maternal and Fetal Health Research Centre, St. Mary’s Hospital, University of Manchester, 5th Floor, Oxford Road, Manchester, M13 9WL UK; 2 St. Mary’s Hospital, Manchester, UK

**Keywords:** Viral infection, Epidemiology, Risk factors

## Abstract

There is conflicting evidence regarding the effect of coronavirus disease (COVID-19) in pregnancy. Risk factors for COVID-19 overlap with risk factors for pregnancy complications. We aimed to assess the effects of the COVID-19 pandemic and confirmed SARS-CoV-2 infection on pregnancy outcomes. A retrospective interrupted time-series and matched cohort analysis was performed. Singleton pregnancies completed between 1st January 2016 and 31st January 2021 were included. Trends in outcomes were analysed over time. Modelled COVID-19 transmission data were applied to deliveries since 1st January 2020 to assign a risk of COVID-19 to each pregnancy, and incorporated into a regression model of birthweight. Confirmed COVID-19 cases were matched to controls delivered in the pre-pandemic period, and maternal and neonatal outcomes compared. 43,802 pregnancies were included, with 8343 in the model of birthweight. There was no increase in the risk of stillbirth (*p* = 0.26) or neonatal death (*p* = 0.64) during the pandemic. There was no association between modelled COVID-19 attack rate (%) in any trimester and birthweight (first trimester *p* = 0.50, second *p* = 0.15, third *p* = 0.16). 214 COVID-positive women were matched to controls. Preterm birth was more common in symptomatic cases (14/62, 22.6%) compared to asymptomatic cases (9/109, 8.3%, *p* = 0.008) and controls (5/62, 8.1%, *p* = 0.025). Iatrogenic preterm birth was more common in cases (21/214, 9.8%) than controls (9/214, 4.2%, *p* = 0.02). All other examined outcomes were similar between groups. There was no significant impact of COVID-19 on the examined birth outcomes available. Symptomatic COVID-19 should be considered a risk factor for preterm birth, possibly due to an increase in iatrogenic deliveries for maternal indications.

## Introduction

Since the first reports of pneumonia of unknown cause emerged from Wuhan^1^ in December 2019 and the subsequent identification of the etiological agent as a novel coronavirus^2,3^ now termed SARS-CoV-2, there have been over 180 million cases of Coronavirus disease (COVID-19) and over 4 million attributable deaths globally^4^. The UK has borne a significant proportion of the global burden of COVID-19, with the seventh highest number of cumulative cases at the time of writing^4^.

As our understanding of SARS-CoV-2 infection has developed, concerns have been raised about the effect of COVID-19 in pregnancy. Limited previous experience with SARS-CoV-1 and MERS-CoV showed an increased risk of adverse pregnancy outcomes including preterm birth, pre-eclampsia and perinatal death^5^. The association of COVID-19 with an unfavorable CD4+ T-cell phenotype raised concerns about disordered implantation and placentation^6^, and the possibility of subsequent fetal growth restriction (FGR), pre-eclampsia and other consequences of placental dysfunction. COVID-19 is also associated with a profound prothrombotic state, and in particular with the formation of immunogenic thrombi in the microvasculature^7^. This not only increases the risk of venous thromboembolism^8^ but has been reported by some investigators to lead to high rates of placental fetal and maternal vascular malperfusion^9^, though a subsequent controlled study of placental pathology showed no specific pattern of pathologic features associated with COVID-19^10^. SARS-CoV-2 achieves cell entry via binding to the ACE2 receptor^3^ which is expressed in the syncytiotrophoblast and cytotrophoblast^11^, and as such provides a plausible mechanism for placental infection and vertical transmission to the fetus.

Reports of pregnancy outcomes following COVID-19 have been conflicting. A recent overview of systematic reviews^12^ highlighted wide variation in reported outcomes, with the rate of preterm delivery varying from 14.3 to 61.2% and neonatal death from 0 to 11.7%. A large meta-analysis^13^ reported an increased risk of caesarean section, low birth weight and preterm birth in pregnant women with COVID-19 compared to those without, and found relatively high rates of presumed vertical transmission. Prospective registry studies have shown a more modest effect of maternal COVID-19, with an increased rate of preterm birth but no significant effect on fetal growth, neonatal outcomes or stillbirth^14^. This has not been replicated in low income settings, with significant increases in stillbirth rate and neonatal mortality associated with the national lockdown period in Nepal^15^. National data from the UK^16^ have shown an increase in iatrogenic preterm birth in symptomatic COVID-19, with largely good outcomes and a high proportion of affected women from Black, Asian and “other” minority ethnic groups. Most studies to date have lacked a control group, or compared outcomes to an unmatched historical cohort, and thus may overestimate the effect of COVID-19 due to the overlap between risk factors for COVID-19^16^ and pregnancy complications, including obesity^17^ and minority ethnicity^18,19^.

We aimed to describe the effect of the COVID-19 pandemic on pregnancy outcomes at a population level in a multi-ethnic cohort in England. We also aimed to further examine the effect of confirmed COVID-19 infection on pregnancy outcomes compared to matched controls.

## Materials and methods

Singleton pregnancies booked and completed beyond 16 weeks gestation at a large tertiary maternity unit in North West England between 1st January 2016 and 31st January 2021 were included. The catchment population includes the local inner-city population, the surrounding conurbation and complex pregnancies referred from across the North West, which was unchanged during the pandemic. Patients under the age of 16 years at delivery were excluded. Routinely collected clinical data including demographic data, medical history, obstetric history, SARS-CoV-2 RNA RT-PCR results and maternal and neonatal outcome data were analysed retrospectively. This study was approved by the Health Research Authority UK (HRA), reference 21/HRA/2377. As this retrospective analysis used routinely collected data informed consent was waived by the HRA. All research was performed in accordance with relevant guidelines/regulations.

Hypertension was defined as a clinical diagnosis of hypertension or blood pressure > 140/90 mmHg at booking. Maternal diabetes was defined as pre-existing type 1 or type 2 diabetes mellitus at booking, or attendance at a diabetic antenatal clinic during pregnancy. The COVID era was defined as deliveries from 17th February 2020 onwards, and the pre-COVID era as deliveries up to and including 16th February 2020, during which time community COVID incidence was negligible in North West England according to a published model^20^.

### Time-series analysis

We evaluated the rates of stillbirth (defined as a baby delivered at or after 24 + 0 weeks gestation with no signs of life) and neonatal death (defined as a liveborn baby delivered at or after 20 + 0 weeks gestation that died before 28 completed days after birth) over time. Terminations of pregnancy were excluded from further analysis. Rates of pre-term birth at < 37 weeks gestation and < 34 weeks gestation were compared in the pre-COVID and COVID eras. Birth weight z-scores were calculated to adjust birth weight for gestational age; a third degree polynomial function was fitted to the 50th centile of estimated fetal weight (EFW) using WHO fetal growth charts data^21^, and z-scores calculated assuming a coefficient of variation of 11.3% between 20 + 0 and 29 + 6 weeks gestation, and 11.8% beyond 30 + 0 weeks. Centile limits were defined using z-scores, and fetal growth restriction (FGR) was defined as birth weight < 3rd centile at any gestation, or birth weight < 10th centile AND born < 34 weeks gestation^22,23^. Caesarean section rate was examined over time.

In order to account for under-ascertainment of COVID positive cases, estimates of COVID-19 incidence in North West England from a living transmission model of the COVID-19 pandemic^20^ were used to calculate the COVID-19 attack rate (% of population contracting COVID-19 during a specified time interval) for each pregnancy during each trimester since 1 January 2020. The first trimester was defined as 0 to 12 + 6 weeks gestation, the second as 13 + 0 to 27 + 6 weeks, and the third as 28 + 0 weeks until delivery. The attack rate in each trimester was then used as a proxy for SARS-CoV-2 exposure in the community and incorporated into a multivariate linear regression model of birth weight.

### Matched cohort analysis

COVID positive cases were identified as those women who had a nasopharyngeal swab positive for SARS-CoV-2 RNA using real-time polymerase chain reaction (RT-PCR) at our center between conception and 3 days postnatal. Risk factors for a positive swab were assessed using multivariate logistic regression.

Cases were matched with controls who had delivered between 1 January 2019 and 16 February 2020. Exact matching without replacement was performed on hypertensive status at booking, diabetic status in pregnancy, primiparity or multiparity, age at delivery (more or less than 35 years), BMI category (< 18.5, 18.5–25, 25–30, 30–40, > 40 kg/m^2^) and ethnicity. Where multiple matches were identified, the closest match was selected based on the month of conception in order to account for seasonality, and any ties were broken randomly.

Maternal and neonatal outcomes were compared between COVID positive cases and controls. Birth outcomes were recorded including neonatal and maternal length of stay, maternal critical care admission, neonatal intensive care unit (NICU) admission and estimated blood loss. Cord blood acid/base status data were collected where available and used as a surrogate of neonatal condition; samples were included for analysis where paired arterial and venous samples were taken and the difference between arterial and venous pH exceeded 0.02. Cases were excluded from fetal growth analysis where the positive test occurred within 14 days of delivery. Inferential tests statistics are not reported where the number of events in either group was < 5.

### Statistics

Statistical analyses were performed in Excel (Microsoft Corporation, USA) and Stata 14 (StataCorp, USA). Descriptive statistics are reported as number (percentage) for categorical data, mean ± SD for parametric data, and median (IQR) for non-parametric data. Comparison of means was performed using a t-test for parametric data (paired where appropriate). For non-parametric data, comparison of mean ranks was performed using the Mann–Whitney U test, or Wilcoxon signed-rank test when paired. Comparison of proportions was performed using the chi-squared test. Logistic regression was used to assess the risk of binary outcomes, and data are presented as an odds ratio with 95% confidence interval. Linear regression was used to assess linear relationships between variables, and data are presented as a coefficient with 95% confidence interval. In the time series analysis, regression analysis was time-adjusted to account for trends independent of the pandemic. Significance was set at a *p* value of 0.05. A priori sample size calculations were not performed due to the retrospective nature of this analysis, and all eligible consecutive cases were included.

## Results

After exclusion of 26 deliveries with a maternal age of less than 16 years, 43,802 deliveries from 37,157 individual women were included. 36,288 pregnancies were completed in the pre-COVID era, and 7,514 in the COVID era (17 February 2020 to 31 January 2021).

### Demographics

Demographic data for the included pregnancies are presented in Table 1.

**Table 1:** Demographic data for included pregnancies.

	Pregnancies (n = 43,802)
Maternal age (years)	30 (26–34)
**Ethnicity**	
Asian	9938 (22.7%)
Black	5675 (13.0%)
Mixed	1121 (2.6%)
Other	2627 (6.0%)
White	24,127 (55.1%)
Not recorded	314 (0.7%)
Parity	1 (0–2)
BMI (kg/m^2^)	26.3 ± 5.8
Diabetes in pregnancy	2625 (6.0%)
Hypertension at booking^a^	1626/43,631 (3.7%)
**Sex of baby**	
Male	22,364 (51.1%)
Female	21,406 (48.9%)
Indeterminate	32 (0.1%)

Data are presented as median (interquartile range), mean ± standard deviation, or number (percentage) as appropriate for data type and distribution.

^a^171 pregnancies did not have a blood pressure measurement recorded at booking.

### Time-series analysis

#### Perinatal deaths

**Figure 1:**
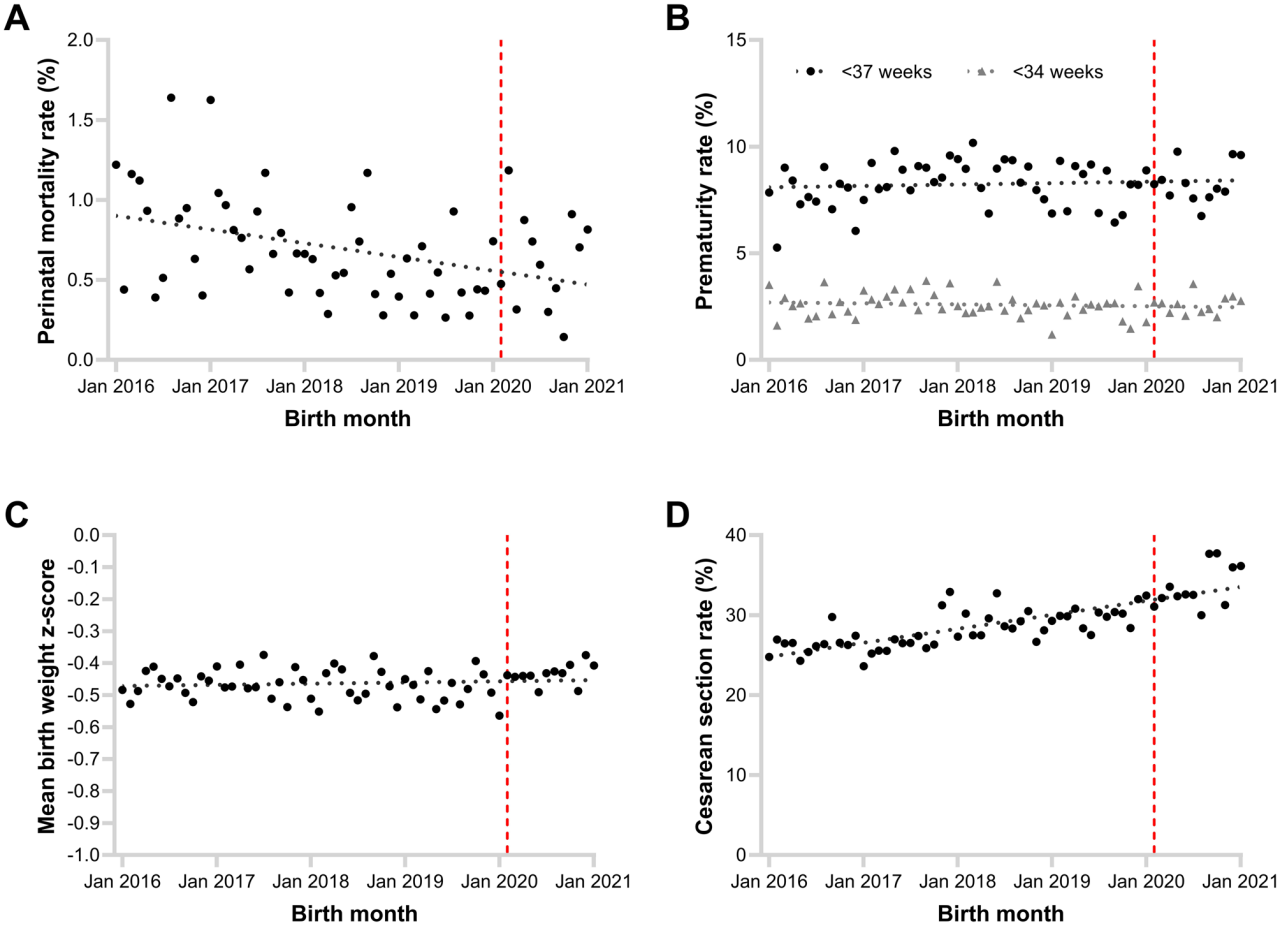
Changes in pregnancy outcomes over time. Scatter plots show (**A**) extended perinatal mortality rate including stillbirth and neonatal death, (**B**) rates of preterm birth at < 37 and < 34 weeks, (**C**) birth weight adjusted for gestation using z-score, and (**D**) caesarean section rate by month of birth between January 2016 and January 2021. Dotted lines are simple linear regressions of the presented data points, and the vertical dashed lines represent the start of the COVID era on 17 February 2020.

After excluding 47 terminations, there were 116 neonatal deaths and 179 stillbirths (0.26% and 0.41% respectively). There was no significant change in the risk of stillbirth (OR 0.78, 95% CI 0.51–1.20, *p* = 0.26) or neonatal death (OR 0.89, 95% CI 0.54–1.47, *p* = 0.64) in the COVID era compared to the pre-COVID era (Fig. 1A).

#### Preterm birth

42 deliveries were excluded from gestation analysis as they had an implausible gestation at birth of more than 300 days.

3,215 (7.4%) babies were born before 37 weeks, and 1,030 (2.4%) before 34 weeks. There was no significant change in the risk of preterm birth at less than 37 weeks (OR 1.00, 95% CI 0.91–1.10, *p* = 0.93) or at less than 34 weeks (OR 1.00, 95% CI 0.85–1.18, *p* = 0.97) in the COVID era compared to the pre-COVID era (Fig. 1B).

#### Birth weight

572 deliveries were excluded from birth weight analysis due to missing birth weight or a gestation at birth of less than 20 weeks.

Mean birth weight was 3,284 g ± 577 g, which equates to a mean birth weight z-score of -0.46 ± 1.10 as compared to the WHO fetal growth charts^21^. There was no significant change in birth weight z-score over time (*p* = 0.50), however there was a trend towards an increase in z-score in the COVID era (Mean difference 0.04, 95% CI 0.00–0.07, *p* = 0.054) compared to the pre-COVID era (Fig. 1C).

3,548 (8.2%) babies were identified as having FGR. There was no significant change in the risk of FGR (OR 0.98, 95% CI 0.89–1.07) in the COVID era compared to the pre-COVID era.

#### Caesarean section rate

The overall caesarean section rate was 29.0%. There was a steady increase in caesarean section rate over time, with an average increase of 1.6% per calendar year excluding January 2021. Adjusting for this increase over time, the risk of caesarean section remained significantly higher during the COVID era (OR 1.11, 95% CI 1.03–1.19, *p* = 0.006) (Fig. 1D).

#### Incorporating modelled transmission data

8523 deliveries between 1 January 2020 and 31 January 2021 were included in this analysis. During the entire COVID era, the COVID-19 attack rate was estimated at 30% (95% CI 25 to 40%) in NW England^20^, i.e. approximately 30% of the population of NW England had contracted COVID-19 as of 7 February 2021.

**Figure 2:**
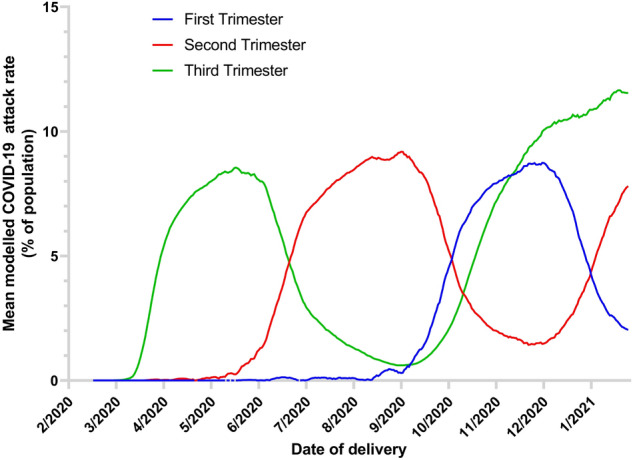
Changes in modelled cumulative COVID-19 exposure during pregnancy. A graph illustrating the average modelled risk of COVID-19 (expressed as attack rate, or the % of population contracting COVID-19 during a specified time interval, calculated from modelled transmission data^20^) in each trimester of pregnancy, plotted against date of delivery between 17 February 2020 and 31 January 2021. The first trimester was defined as 0 to 12 + 6 weeks gestation, the second as 13 + 0 to 27 + 6 weeks, and the third as 28 + 0 weeks to delivery. Women exposed to COVID-19 in early pregnancy only delivered in the latter part of the study period.

The median attack rate in the first trimester of each pregnancy was 0% (0–2.9%), 1.2% (0–6.3%) in the second trimester, and 2.5% (0.3–7.8%) in the third trimester. This reflects that women exposed to COVID-19 in the first and second trimesters of pregnancy did not start delivering until later in the study period, and therefore the average exposure in the third trimester is highest. Change in modelled COVID-19 exposure in each trimester over time is illustrated in Fig. 2.

A multivariate linear regression was calculated to predict birth weight based on gestation at birth; maternal height, weight, parity, and ethnicity; neonatal sex; and the COVID-19 attack rate in each trimester. 180 deliveries were excluded due to missing birth weight, missing height or weight at booking, indeterminate neonatal sex or termination of pregnancy. Regression statistics are reported in Table 2. Nine women had more than one baby during this period, and accounting for this had no effect on the reported coefficients (data not shown).

**Table 2:** Regression statistics for linear regression model of birth weight (g).

Predictor	Coefficient	95% confidence interval	*p* value
Gestation at birth (days)	26.3	25.7 to 26.9	< **0.001**
Height at booking (cm)	7.9	6.5 to 9.2	< **0.001**
Weight at booking (kg)	4.9	4.3 to 5.4	< **0.001**
Parity	26.1	20.0 to 32.1	< **0.001**
Female baby	− 111.4	− 128.3 to − 94.4	< **0.001**
**Ethnicity**			
White	0.0 (ref)		
Black	− 88.2	− 114.0 to − 62.4	< **0.001**
Asian	− 86.0	− 107.8 to − 64.3	< **0.001**
Mixed	− 121.9	− 176.3 to − 67.5	< **0.001**
Other	− 15.0	− 48.3 to 18.2	0.38
Not recorded	− 26.6	− 124.2 to 70.9	0.59
**COVID-19 attack rate**^**a**^** (%)**			
First trimester	1.1	− 2.0 to 4.2	0.50
Second trimester	1.9	− 0.7 to 4.4	0.15
Third trimester	2.0	− 0.8 to 4.7	0.16

The COVID-19 attack rate (% of population contracting COVID-19 during a specified time interval) was calculated using modelled transmission data^20^ and is used here as a proxy for COVID-19 exposure in each trimester.

Significant values are in bold.

Increasing gestation at birth and maternal height, weight and parity were all associated with an increased birth weight (*p* < 0.001 for all). Female neonatal sex and Black, Asian or Mixed ethnicities were associated with a reduced birth weight (*p* < 0.001 for all). Adjusting for the above, there was no significant association between modelled COVID-19 incidence (expressed as attack rate) in any trimester and birth weight (Table 2).

Alternative regression models are reported in the supplementary material, using a polynomial fit of gestation to birth weight (Table S1) and a linear regression of birth weight z-score (Table S2). Effect sizes were concordant in the three models.

### Matched cohort analysis

Of 7514 deliveries in the COVID era, 218 women were identified as having a positive RT-PCR swab for SARS-CoV-2 between conception and three days postnatal. Considering only those deliveries during the COVID era, risk factors for a positive swab are shown in Table 3. Significant risk factors include maternal BMI > 35 kg/m^2^, and Black or Asian ethnicities.

**Table 3:** Risk factors for a positive RT-PCR swab for SARS-CoV-2.

Risk factor	Odds ratio	95% CI	*p* value
Maternal age > 35 years	0.75	0.54–1.05	0.09
BMI > 35 kg/m^2^	1.71	1.14–2.55	**0.009**
**Ethnicity**			
White	1.0 (ref)^a^		
Black	1.62	1.10–2.37	**0.01**
Asian	1.58	1.14–2.20	**0.006**
Mixed	0.45	0.11–1.86	0.27
Other	1.50	0.92–2.46	0.11
Not recorded	1.43	0.34–5.97	0.62

Significant values are in bold.

^a^For ethnicity, the modal group was used as a reference to calculate odds ratios.

One pregnancy was excluded due to termination for genetic abnormality, and three pregnancies were unable to be matched, leaving 214 matched pairs for further analysis. 91 women (42.5%) delivered at least 14 days after a positive swab and were included in analysis of fetal growth. Placental histopathology was available in 25 cases (15 preterm and 10 term births) and is reported in Table S3.

Baseline characteristics and outcomes are reported in Table 4.

**Table 4:** Baseline characteristics and outcome data in COVID-19 cases and matched controls.

	COVID-19 cases (n = 214)	Controls (n = 214)	*p* value
**Baseline Characteristics**			
Maternal age (years)	30 (26–34)	30 (26–34)	0.91
**Ethnicity**			
Asian	65 (30.4%)	65 (30.4%)	–
Black	37 (17.3%)	37 (17.3%)	–
Mixed	2 (0.9%)	2 (0.9%)	–
Other	19 (8.9%)	19 (8.9%)	–
White	89 (41.6%)	89 (41.6%)	–
Not recorded	2 (0.9%)	2 (0.9%)	–
Parity	1 (0–2)	1 (0–2)	0.30
BMI (kg/m^2^)	27.3 ± 6.1	27.1 ± 6.3	0.46
Diabetes in pregnancy	17 (7.9%)	17 (7.9%)	–
Hypertension at booking	7 (3.3%)	7 (3.3%)	–
**Sex of baby**			
Male	119 (55.6%)	118 (55.1%)	0.60
Female	94 (43.9%)	96 (44.9%)	
Indeterminate	1 (0.5%)	0 (0.0%)	
**Trimester at diagnosis**			
First	3 (1.4%)	–	–
Second	25 (11.7%)	–	–
Third	186 (86.9%)	–	–
**Neonatal Outcomes**			
**Mode of delivery**			
Spontaneous vaginal	105 (49.1%)	114 (53.3%)	0.70
Forceps	26 (12.2%)	30 (14.0%)	
Ventouse	10 (4.7%)	8 (3.7%)	
Emergency CS	46 (21.5%)	41 (19.2%)	
Elective CS	27 (12.6%)	21 (9.8%)	
Gestation at delivery (days)	275 (269–281)	278 (271–284)	**0.002**
**Preterm birth**			
< 37 weeks	27 (12.6%)	18 (8.4%)	0.16
< 34 weeks	7 (3.3%)	3 (1.4%)	0.20
Iatrogenic (< 37 weeks)	21 (9.8%)	9 (4.2%)	**0.02**
Placental abruption	3 (1.4%)	1 (0.5%)	N/A^a^
Stillbirth	1 (0.5%)	0 (0.0%)	N/A^a^
Neonatal death	2 (0.9%)	2 (0.9%)	N/A^a^
NICU admission	25 (11.7%)	30 (14.0%)	0.48
NICU length of stay (days)	6 (3–12) (n = 25)	3.5 (2–8) (n = 30)	0.25
Cord arterial pH	7.18 ± 0.09 (n = 82)	7.18 ± 0.08 (n = 98)	0.49
Cord arterial base deficit	7.0 ± 3.7 (n = 82)	7.4 ± 3.6 (n = 98)	0.75
**Maternal Outcomes**			
Estimated blood loss (ml)	400 (300–700)	450 (300–700)	0.39
Maternal length of stay (days)	2 (1–4)	2 (1–4)	0.81
Pre-eclampsia	6 (2.8%)	8 (3.7%)	0.60
HDU admission	12 (5.6%)	12 (5.6%)	1.00
ICU admission	4 (1.9%)	0 (0.0%)	N/A^a^

	COVID-19 Cases (n = 91^b^)	Controls (n = 91^b^)	Significance (*p* value)
**Growth**			
Birth weight (g)	3244 ± 616	3351 ± 584	0.12
Birth weight z-score	− 0.29 ± 1.20	− 0.36 ± 1.16	0.66
Fetal growth restriction	8 (8.8%)	9 (9.9%)	0.80

Data are presented as median (IQR), mean ± SD, or number (%) as appropriate for data type and distribution. Comparisons were performed using the chi-squared test, paired and unpaired t-tests, Wilcoxon sign-rank test, and Mann–Whitney U test as appropriate.

Significant values are in bold.

*CS* Caesarean section, *HDU* High dependency unit, *ICU* Intensive care unit, *NICU* Neonatal intensive care unit.

^a^Inferential tests statistics are not reported where number of events was less than 5 in both groups.

^b^Only pregnancies where the time between a positive swab and delivery exceeded 14 days are included in analysis of fetal growth.

Though there was no significant difference in the mode of delivery between cases and controls (*p* = 0.70), a larger proportion of emergency caesarean sections were performed for maternal disease in women with COVID-19 (11/46, 23.9%) compared to controls (2/41, 4.9%) (*p* = 0.01). This includes five caesarean sections performed due to worsening COVID-19, four for pre-eclampsia, and two for maternal medical disease. There was a significant increase in iatrogenic (caesarean or induced) preterm deliveries in women with COVID-19 (21/214, 9.8%) compared to controls (9/214, 4.2%, *p* = 0.02).

**Figure 3:**
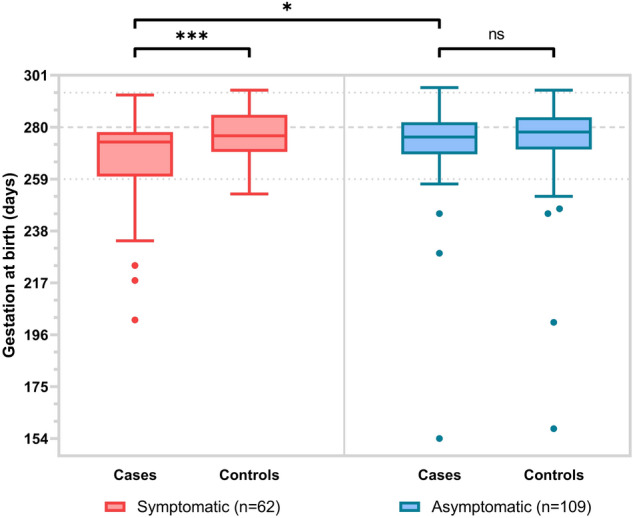
The distribution of gestation at birth for COVID-19 cases and matched controls, grouped by symptom status. Boxes represent the median and interquartile range, whiskers show the Tukey fences and closed circles show outliers. The dashed reference line is given at 40 + 0 weeks, with dotted lines at 37 + 0 and 42 + 0 weeks gestation. *p < 0.05, ***p < 0.001, *ns* Not significant.

Data were available on symptoms for 171 COVID positive women, with 109 (63.7%) being asymptomatic and 62 (36.3%) symptomatic. Neonatal and maternal outcomes are reported by symptom status in Table S4. Rates of preterm birth at < 37 weeks were higher in women with symptomatic COVID-19 (14/62, 22.6%) compared to asymptomatic women (9/109, 8.3%, *p* = 0.008) and controls (5/62, 8.1%, *p* = 0.025). Distributions of gestation at birth by symptom status in cases and controls are illustrated in Fig. 3. There was no significant difference in mean birth weight z-score between the symptomatic (− 0.37 ± 1.24) and asymptomatic groups (− 0.72 ± 0.73, *p* = 0.83).

32 neonates had a swab for SARS-CoV-2 prior to discharge, 18 of which were performed within 1 day of birth. Of these, 16/18 (88.9%) were negative, and 2/18 (11.1%) were reported as ‘indeterminate’ suggesting very small quantities of SARS-CoV-2 RNA were detected. There were no cases of serious neonatal COVID-19, and no neonatal deaths attributable to COVID-19.

## Discussion

### Principal findings

We found a significant effect of confirmed COVID-19 on preterm birth confined to symptomatic women, although there was no change in rates of preterm birth overall. The risk of preterm birth in asymptomatic COVID-19 was similar to that of the uninfected population. Increases in the overall caesarean section rate, the number of emergency caesarean sections for maternal indications, and the rate of iatrogenic preterm birth in women with COVID-19 suggest the increase in preterm birth is attributable to obstetric practice. This may represent an increase in indicated early delivery to aid management of maternal COVID-19, or symptomatic COVID-19 may lower the clinician’s threshold to intervene for other reasons. It is worth noting a recent international meta-analysis found significant heterogeneity within these outcomes^24^, and whilst we saw an increase in preterm birth there seems to be significant global variation in how obstetric practice has changed during the pandemic.

There was no significant effect of COVID-19 on fetal growth, including in a large analysis incorporating modelled community incidence. We also saw no significant increase in rates of pre-eclampsia in women with COVID-19, which also suggests COVID-19 is not having a profound effect on placental function, although this study is not powered to detect a less than threefold increase in the incidence of pre-eclampsia.

Neonates born to mothers with COVID-19 seemed to do as well as their matched counterparts, with no increase in rates of admission to neonatal intensive care or in length of stay for those that were admitted. Neonatal acid base status was also equivalent between the groups, which provides further reassurance that COVID-19 does not increase the risk of adverse perinatal outcomes. Equally, there was no increase in maternal length of stay or estimated blood loss in those with confirmed COVID-19, suggesting that COVID-19 is not having a serious effect on maternal outcomes in labor and delivery, though this matched cohort analysis is underpowered to detect smaller differences in these outcomes.

### Results in context

COVID-19 was significantly more likely to affect Black and Asian women in line with national data^16^, and obese women also seemed to have a higher risk of a positive swab. This may be confounded by an increased risk of severe disease in obesity^25^ and therefore obese women are likely over-represented in those presenting for a test. Most women with a positive test were asymptomatic, in keeping with reports^26^ that pregnant women are significantly less likely than the general population to show symptoms of COVID-19.

We saw no effect of COVID-19 on fetal growth, either in women with confirmed infection or in the cohort as a whole. This is consistent with prospective data from the UK and US^14^, and adds to growing evidence that concerns around fetal growth restriction caused by COVID-19 may be unfounded.

There was no evidence of an increase in perinatal death rates in contrast to reports from elsewhere in the UK^27^. The number of perinatal deaths at our center are small however, and a larger review of national data may be able to more conclusively determine any effect of COVID-19 on stillbirth and neonatal death. Preprint data from the national UK Obstetric Surveillance System has reported no significant increase in perinatal mortality in pregnancies affected by COVID-19^16^, albeit with wide confidence intervals.

It may be that increases in perinatal deaths seen in low income settings are due to reduced access to healthcare during lockdown rather than a biological effect of COVID-19, or conversely that a focus on reduced fetal movements in higher income settings encourages women to present early and avoid fetal demise.

We found some evidence of possible vertical transmission, with two cases where small amounts of SARS-CoV-2 RNA was detected in a nasopharyngeal swab shortly after delivery. It is unclear whether this was truly vertically transmitted or transferred during delivery. Regardless, there were no cases of severe neonatal COVID-19, consistent with previous data showing vertical transmission and neonatal COVID-19 are uncommon^28^.

Various histopathological lesions were identified in those placentas sent for examination, however the incidence of these is likely to be overestimated due to selection bias and a lack of blinding. These lesions can be identified in up to 78% of normal term pregnancies^29^, and therefore may not be clinically significant.

### Clinical implications

The only significant effect of COVID-19 in pregnancy identified here was an increase in preterm birth in symptomatic women. Hence, women with asymptomatic COVID-19 may not need additional monitoring. We saw no effect on fetal growth, suggesting fetal growth surveillance, currently recommended in the UK following COVID-19^30^, may be unnecessary.

### Research implications

Larger studies are needed to further investigate the relationship between COVID-19 and rare events, including the risk of perinatal death, pre-eclampsia and placental abruption. Pooling data in metanalyses may assist in detecting signals in these outcomes.

### Strengths and limitations

Strengths of this study include its large size and carefully matched control group. Unfortunately, as for many contemporaneous studies, there is likely significant under-ascertainment of COVID-19 cases, with 218 cases identified where we might expect around 712–1423 (10–20%) women to have been infected during their pregnancy. This is due in part to introduction of routine screening after the first wave of the pandemic, and also to a large proportion of COVID-19 tests being performed in the community, the results of which are not available to us. It is therefore difficult to assess the true effect of COVID-19 on rare events such as perinatal deaths and placental abruption. We attempted to account for this using modelled transmission data, however this does not account for differences in behavior between pregnant and non-pregnant people. If the model systematically overestimated COVID exposure in pregnant people, the effect size on birthweight might be underestimated, however we wouldn’t expect the significance to change. As a retrospective analysis our results are also limited by available data, and as such we were not able to assess the impact of smoking status on outcomes as this was not reliably recorded, and symptom data were not available in all women. More granular outcome data examined in the matched cohort analysis were not coded at a population level, including rates of pre-eclampsia and estimated blood loss.

## Conclusions

Symptomatic COVID-19 is associated with preterm birth, which may be due to an increase in iatrogenic deliveries for maternal indications. However, there seems to be no effect of COVID-19 on fetal growth, and maternal and neonatal outcomes are comparable to those seen in women without COVID-19, particularly for asymptomatic women. This is reassuring for expectant mothers and those caring for them; indeed some iatrogenic prematurity may be avoidable in light of this knowledge. We found no evidence of an increase in perinatal deaths associated with the pandemic. These data support continued efforts to prevent COVID-19 in pregnancy, however additional fetal growth surveillance following COVID-19 may be unnecessary.

## Supplementary Information

Supplementary Information.

## Data Availability

The datasets generated during and/or analysed during the current study are available from the corresponding author on reasonable request.
