# Supplementary material for: The impact of COVID-19 on pregnancy outcomes in a diverse cohort in England
Source: Sci Rep. 2022 Jan 18;12:942. doi: 10.1038/s41598-022-04898-5 (PMC8766432; doi:10.1038/s41598-022-04898-5)
Supplement: Supplementary file 1 — Supplementary Information. [file 41598_2022_4898_MOESM1_ESM.docx]

**TITLE:** The impact of COVID-19 on pregnancy outcomes in a diverse cohort in England

**AUTHORS**: Michael WILKINSON, MBBS^1^, Edward D JOHNSTONE, PhD MRCOG^1^, Louise E SIMCOX, MD MRCOG^2^, Jenny E MYERS*, PhD MRCOG^1^

**AFFILIATIONS:**
^1^Maternal & Fetal Health Research Centre, University of Manchester, UK
^2^St. Mary's Hospital, Manchester, UK

**DISCLOSURES:** The authors report no conflict of interest.
**SOURCES OF FUNDING:** The authors received no specific funding for this work.

**CORRESPONDING AUTHOR:**

Professor Jenny Myers
Email: [jenny.myers@manchester.ac.uk](mailto:jenny.myers@manchester.ac.uk)
Tel: +44 161 701 6963
Address: Maternal & Fetal Health Research Centre, 5^th^ Floor, St Mary’s Hospital, Oxford Road, Manchester M13 9WL

**Table S1.** Regression statistics for fractional polynomial regression model of birth weight

| Predictor | | | Coefficient (g) | 95% conf. interval (g) | p-value |
| --- | --- | --- | --- | --- | --- |
| Gestation at birth (days)^a^ | | Term 1 𝑥^-2^ | 2.09e+09 | 1.96e+09 to 2.21e+09 | **<0.001** |
|  |  | Term 2 𝑥^-2^ | -4.59e+08 | -4.85e+08 to -4.33e+08 | **<0.001** |
| Height (cm) | | | 7.9 | 6.5 to 9.3 | **<0.001** |
| Weight (kg) | | | 4.9 | 4.3 to 5.4 | **<0.001** |
| Parity | | | 25.8 | 19.7 to 31.9 | **<0.001** |
| Female baby | | | -112.1 | -129.0 to -95.2 | **<0.001** |
| Ethnicity | White | | 0.0 (ref) |  |  |
|  | Black | | -89.8 | -115.5 to -64.1 | **<0.001** |
|  | Asian | | -87.1 | -108.8 to -65.4 | **<0.001** |
|  | Mixed | | -121.3 | -175.5 to -67.1 | **<0.001** |
|  | Other | | -14.2 | -47.4 to 18.9 | 0.40 |
|  | Not recorded | | -30.0 | -127.2 to 67.3 | 0.55 |
| COVID-19 attack rate (%) | First trimester | | 1.0 | -2.1 to 4.1 | 0.52 |
|  | Second trimester | | 1.8 | -0.7 to 4.3 | 0.16 |
|  | Third trimester | | 1.8 | -0.9 to 4.6 | 0.19 |

^a^To account for the non-linear relationship between gestation and birth weight, a fractional polynomial fit was used in the regression model. This had a very small effect on the deviance in the model, with the R^2^ remaining at 0.51.

**Table S2.** Regression statistics for linear regression model of birth weight z-score

| Predictor | | Coefficient | 95% conf. interval | p-value | Effect size at 40 weeks (g)^a^ |
| --- | --- | --- | --- | --- | --- |
| Gestation at birth (days)^b^ | | -0.0006 | -0.002 to 0.0009 | 0.43 | -0.25 |
| Height at booking (cm) | | 0.019 | 0.016 to 0.023 | **<0.001** | +7.9 |
| Weight at booking (kg) | | 0.013 | 0.011 to 0.014 | **<0.001** | +5.4 |
| Parity | | 0.071 | 0.056 to 0.087 | **<0.001** | +29.5 |
| Female baby | | -0.281 | -0.324 to -0.237 | **<0.001** | -116.9 |
| Ethnicity | White | 0.0 (ref) |  |  |  |
|  | Black | -0.231 | -0.298 to -0.165 | **<0.001** | -96.1 |
|  | Asian | -0.209 | -0.265 to -0.153 | **<0.001** | -86.9 |
|  | Mixed | -0.305 | -0.445 to -0.164 | **<0.001** | -126.9 |
|  | Other | -0.035 | -0.121 to 0.051 | 0.42 | -14.6 |
|  | Not recorded | -0.066 | -0.318 to 0.187 | 0.61 | -27.5 |
| COVID-19 attack rate (%) | First trimester | -0.0006 | -0.009 to 0.007 | 0.88 | -0.25 |
|  | Second trimester | 0.005 | -0.001 to 0.012 | 0.12 | +2.1 |
|  | Third trimester | 0.006 | -0.0007 to 0.013 | 0.08 | +2.5 |

^a^To aid interpretation of the coefficients, the effect size is illustrated at 40 weeks gestation assuming a mean birth weight of 3617g and a standard deviation of 416g.
^b^Gestation is included here to account for the relationship between preterm birth and fetal growth restriction

**Table S3.** Placental histopathology in COVID-19 cases

| PLACENTAL PATHOLOGICAL FEATURES | | NUMBER OF PLACENTAS |
| --- | --- | --- |
| Not reported | | 189 |
| Normal | | **1/25 (4%)** |
| Acute inflammatory response | | **7/25 (28%)** |
| Maternal | Acute subchorionitis or chorionitis | 5 |
|  | Acute chorioamnionitis | 5 |
|  | Necrotising chorioamnionitis | 1 |
| Fetal | Funisitis | 4 |
|  | Acute villitis | 1 |
| Chronic inflammatory response | | **8/25 (32%)** |
| Maternal | Chronic chorioamnionitis | 1 |
|  | Chronic villitis | 7 |
|  | Chronic histiocytic intervillositis | 2 |
| Fetal | Lymphocytic fetal vasculitis | 1 |
| Maternal vascular malperfusion | | **16/25 (64%)** |
| Increased syncytial knots | | 8 |
| Intervillous fibrin deposition | | 7 |
| Placental infarction | | 4 |
| Accelerated villous maturation | | 4 |
| Villous hypoplasia | | 2 |
| Decidual arteriopathy | | 2 |
| Fetal vascular malperfusion | | **1/25 (4%)** |
| Villous changes | | 0 |
| Vessel thrombosis | | 1 |
| Delayed villous maturation | | **2/25 (8%)** |

**Table S4.** Pregnancy outcome data in COVID-19 cases and matched controls by symptom status

|  | | | Symptomatic  (n=62) | | | Asymptomatic  (n=109) | | |
| --- | --- | --- | --- | --- | --- | --- | --- | --- |
|  |  |  | **Cases** | **Controls** | **p** | **Cases** | **Controls** | **p** |
| Delivery | Mode of Delivery | Spontaneous vaginal | 32 (51.6%) | 35 (56.5%) | 0.53 | 50 (45.9%) | 56 (51.4%) | 0.58 |
|  |  | Emergency CS | 17 (27.4%) | 11 (17.7%) |  | 21 (19.3%) | 22 (20.2%) |  |
|  |  | Elective CS | 8 (12.9%) | 7 (11.3%) |  | 15 (13.8%) | 9 (8.3%) |  |
|  |  | Forceps | 5 (8.1%) | 8 (12.9%) |  | 13 (11.9%) | 16 (14.7%) |  |
|  |  | Ventouse | 0 (0.0%) | 1 (1.6%) |  | 9 (8.3%) | 6 (5.5%) |  |
|  |  | Breech extraction | 0 (0.0%) | 0 (0.0%) |  | 1 (0.9%) | 0 (0.0%) |  |
| Neonatal Outcomes | Gestation at delivery (days) | | 274 (260-278) | 276.5 (270-285) | **<0.001** | 276 (269-282) | 278 (271-284) | 0.36 |
|  | Preterm birth  (<37 weeks) | | 14 (22.6%) | 5 (8.1%) | **0.03** | 9 (8.3%) | 10 (9.2%) | 0.81 |
|  | Preterm birth  (<34 weeks) | | 5 (8.1%) | 0 (0.0%) | **0.02** | 2 (1.8%) | 2 (1.8%) | 1.00 |
|  | NICU admission | | 11 (17.7%) | 9 (14.5%) | 0.63 | 7 (6.4%) | 16 (14.7%) | **0.047** |
|  | NICU length of stay (days) | | 6 (3-12) (n=11) | 4 (2-6) (n=9) | 0.22 | 12 (3-38) (n=7) | 4.5 (2.5-12.5) (n=16) | 0.20 |
| Maternal | Estimated blood loss (ml) | | 475 (350-750) | 450 (300-700) | 0.47 | 400 (300-700) | 450 (300-700) | 0.65 |
|  | Maternal length of stay (days) | | 3 (2-4) | 3 (2-4) | 0.23 | 2 (1-3) | 2 (1-4) | 0.10 |
| Growth |  | | **Cases**  **(n=37)** | **Controls**  **(n=37)** | **p** | **Cases**  **(n=13)** | **Controls**  **(n=13)** | **p** |
|  | Birth weight z-score | | -0.37 ± 1.24 | -0.32 ± 1.37 | 0.42 | -0.72 ± 0.73 | -0.27 ± 1.01 | 0.09 |
|  | Fetal growth restriction | | 4 (10.8%) | 5 (13.5%) | 0.72 | 2 (15.4%) | 0 (0.0%) | 0.14 |
